# Improving Standardization and Access to Care via Seizure Pathways in the Emergency Department

**DOI:** 10.5811/westjem.48847

**Published:** 2026-01-03

**Authors:** Brian E. Emmert, Cody L. Nathan, James J. Gugger, Kathryn A. Davis, Margaret Provencher, Laura A. Stein, Keith C. Hemmert

**Affiliations:** * Columbia University, Vagelos College of Physicians and Surgeons, Department of Neurology, Division of Epilepsy, New York, New York; † Northwestern Memorial Hospital, Department of Neurology, Division of Epilepsy, Chicago, Illinois; ‡ University of Rochester, Department of Neurology, Rochester, New York; § Perelman School of Medicine at the University of Pennsylvania, Department of Neurology, Division of Epilepsy, Philadelphia, Pennsylvania; || Perelman School of Medicine at the University of Pennsylvania, Department of Emergency Medicine, Philadelphia, Pennsylvania; # Perelman School of Medicine at the University of Pennsylvania, Department of Neurology, Neurohospitalist Division, Philadelphia, Pennsylvania

## Abstract

**Introduction:**

Seizures are one of the most common neurological presentations to an emergency department (ED), often as a first seizure of life or a breakthrough seizure. There is practice variation regarding the diagnostic workup and management for these patient populations. A standardized pathway for emergent evaluation of first seizure of life or breakthrough seizure currently does not exist, resulting in variability in evaluation and timing of outpatient care.

**Methods:**

We created standardized pathways for evaluation and management of patients presenting to the ED with a first seizure of life or breakthrough seizure. These pathways, implemented at a large, quaternary-care hospital system, were utilized on 130 patients presenting with a seizure and compared with all patients with seizure on whom the pathway was not used, between May 2022–October 2023. Outcomes of interest included ED length of stay (LOS), proportion of patients admitted, time to outpatient follow-up, and difference in resource utilization. We compared categorical variables using chi-square test and continuous variables using the Wilcoxon rank-sum test. Equality of variance between the two cohorts was tested using the Levene test.

**Results:**

There was no statistically significant difference between the percentage of male and female patients evaluated via standard-of-care model (45.6% and 49.5%) and those on the pathway (56.9% and 43.1%). The average age of patients was similar between standard-of-care and pathway groups (41 and 39 years, respectively). Median ED LOS was 5.0 (Interquartile range [IQR] 2.9–9.4) hours for standard of care and 4.8 (IQR 3.1–7.0) hours for pathway (*P* = .34), with a significant difference in variability in time for pathway group (*P* < .001). Fewer patients were admitted or observed with pathway use (*P* < .02). Median time to outpatient follow-up was 41.0 days (IQR 17.0–93.0) with standard of care and 23.5 days (IQR 8.0–57.0) with pathway use (*P* < .001). More urinalyses (*P* < .001), drug screens (*P* < .001), alcohol levels (*P* < .001) and computed tomography for first seizures (*P* < .001) were ordered for the pathway group. Fewer magnetic resonance imaging studies were ordered for patients in the breakthrough seizures group using the pathway (*P* < .001).

**Conclusion:**

Standardized pathways to approach seizure presentation in the ED can reduce variability in care, improve time to outpatient neurologic care, and standardize seizure-safety counseling.

## INTRODUCTION

Approximately 11% of the United States population will experience a seizure in their lifetime.^1^ Seizure account for about 1 in 100 emergency department (ED) visits, totalling nearly one million visits between 1993–2003.^1^,^2^ Up to 30% of patients with epilepsy present to the ED with breakthrough seizures, often undergoing repetitive and costly testing, such as neuroimaging, with limited benefits.^3^–^5^ Patients with an established outpatient practitioner have fewer ED visits and admissions, but delays in follow-up contribute to readmissions and fragmented care.^6^,^7^ Standardized pathways can expedite outpatient referal, reduce ED recidivism and improve resource use.^8^ Such pathways have already been shown to improve outcomes in conditions such as stroke, hip fracture, myocardial infarction, and (in Ireland) seizures, by reducing length of stay (LOS) and readmissions, and improving time to follow-up and adherence to guidelines.^9^,^10^

Currently, the American Academy of Neurology (AAN) provides evidence-based guidelines for a first unprovoked seizure in adults, focusing on counseling about antiseizure medication. Such guidelines do not exist for emergent evaluation or management of breakthrough seizures in patients with known prior seizures; however, comprehensive reviews of the evaluation and treatment of patients presenting with seizures have been well published.^11^,^12^ The American College of Emergency Physicians lacks comprehensive guidelines for first or breakthrough seizures.^13^ This gap in standardized ED care, combined with prolonged delays to outpatient neurology follow-up, prompted us to develop seizure evaluation pathways. Our quality improvement (QI) initiative was designed to reduce time to outpatient follow-up with a neurologist, shorten ED LOS, and standardize care, demonstrating that seizure pathways can be successfully implemented in a large, academic center to optomize patient outcomes.

## METHODS

**Figure 1:**
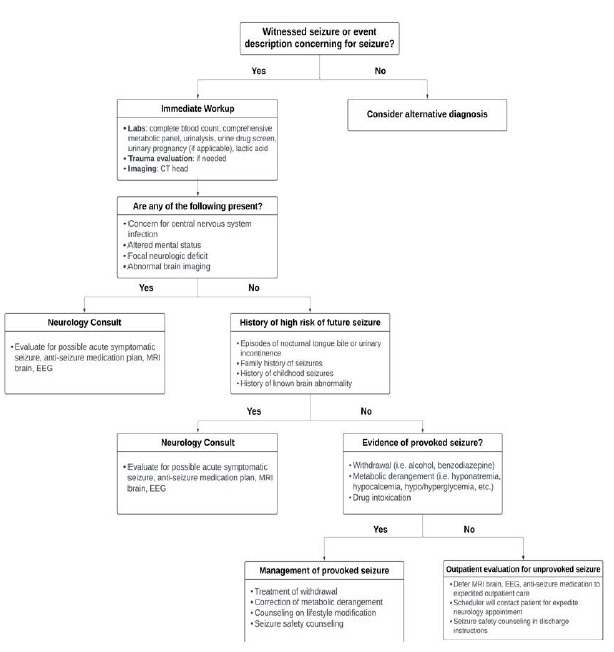
Pathway template for first seizure of life, demonstrating suggested workflow for patients presenting to the emergency department with a first seizure of life. *EEG*, electroencephalogram; *MRI*, magnetic resonance imaging.

**Figure 2:**
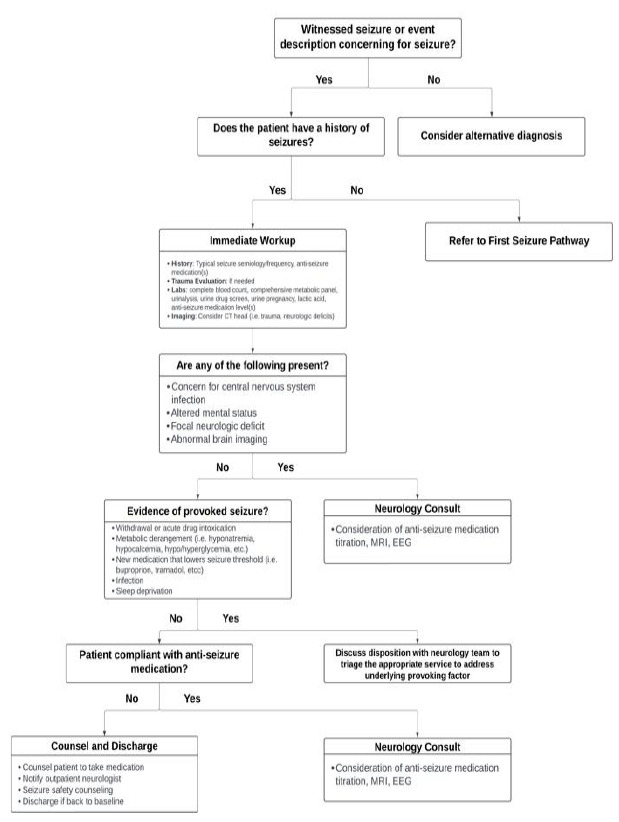
Pathway template for breakthrough seizure, demonstrating suggested workflow for patients presenting to the emergency department with a breakthrough seizure. *EEG*, electroencephalogram; *MRI*, magnetic resonance imaging.

A multidisciplinary team of epileptologists, neurohospitalists, and emergency physicians at a large, urban, multihospital academic center (Level 4 epilepsy center), where lab testing, imaging, and electroencephalogram (EEG) was available 24/7, developed ED clinical pathways for first seizure of life and breakthrough seizures using the Medical Research Council framework.^14^ Pathways were based on AAN best practice guidelines and expert consensus when guidelines were lacking.^11^–^13^ The pathways outlined suggested immediate workup (labs and imaging), “red flag” findings prompting urgent neurologic consultation, indications for expedited EEG/magnetic resonance imaging (MRI), and a structured process for scheduling outpatient neurology follow-up (Figures 1–2). The pathways were designed as guidance for emergency physicians rather than mandatory orders. The pathways were reviewed by senior epileptologists and emergency physicians. The study was granted institutional review board exemption.

**Population Health Research Capsule**

What do we already know about this issue?

*Diagnostic and management approaches to first and breakthrough seizures vary substantially, reflecting the lack of a standardized clinical evaluation pathway*.

What was the research question?

*We implemented emergency department (ED) pathways for first and breakthrough seizures to reduce length of stay (LOS) and improve neurology follow-up*.

What was the major finding of the study?

*Pathway use led to decreased median time to outpatient follow-up (17.0 vs. 41.0 days, P < .001) and variability in LOS (P < .001)*.

How does this improve population health?

*Standardized ED seizure pathways can reduce variability in care, improve time to outpatient neurologic care, and standardize safety counseling*.

Using published literature and expert opinion, the workgroup also developed standardized discharge materials on seizure safety, provoking factors, and first aid for use in the ED (Supplement).^15^ Pathways and discharge literature were published on the health system intranet, and an electronic health record-based (EHR) order set was developed to facilitate recommended testing and expedited follow-up. Patients with existing neurology providers were referred back to them, while others where scheduled with the next available epilepsy advanced practice provider, general neurologist, or epileptologist. Studies that would not result during the ED stay were forwarded to the patient’s existing neurologist or primary care physician. Emergency department faculty across the health system were educated on the existence and use of the pathways. Implementation began in May 2022. We reviewed pathway utilization in October 2022, followed by re-education of staff, which increased use fourfold.

One year after implementation (June 2023), we queried the EHR for all ED seizure presentations (*International Classification of Diseases, 10^th^ Rev* [*ICD-10*] codes including the words “Seizure,” “Epilepsy,” “Convulsion,” “Post-ictal state,” “First time Seizure,” “Breakthrough seizure”) and divided them into standard-of-care and pathway groups. The pathway group was further subdivided into breakthrough seizure and first seizure of life; the non-pathway group could not be subclassified due to limitations with *ICD-10* sampling (ie, limited use of specific codes). Pathway use was clinician-dependent to maintain natural workflow, influenced by frequency of ED presentation, patient complexity, and clinician familiarity with the pathway. Outcomes included ED LOS, admission rates, time to outpatient follow-up, and resource utilization. We compared categorical variables using chi square tests, continuous variables with Wilcoxon rank-sum test, and variance with the Levene test. Analyses were performed using Stata SE 13.1 for Windows (StataCorp, LLC, College Station, TX).

## RESULTS

Across three hospitals, 2,664 patient encounters were identified. The pathways were used in 130 encounters (67 breakthrough seizure, 63 first seizure). Patient demographics are outlined in Table 1. Median LOS was similar between groups: 5.0 hours (Interquartile range [IQR] 2.9–9.4) for standard care vs 4.8 hours (IQR 3.1–7.0) for pathways (*P* = .34). Variability in LOS was lower for pathway use (90^th^ percentile: 11.7 hours for pathway vs 24.4 hours for standard of care, *P* < .001).

No pathway patients were admitted, compared to 120 in the standard-of-care group (*P* < .001). Overnight stays (admission plus observation) occurred in 6.2% of pathway patients vs 12.3% of standard-of-care patients (*P* = .02). Median time to outpatient neurology/epilepsy follow-up was shorter with pathways: 23.5 days (IQR 8–57) vs 41.0 days (IQR 17.0–93.0) (*P* = .001). Subgroup analysis showed 24.0 days (IQR 9.0–62.0) for first seizure of life (*P* = .047) and 16.0 days (IQR 7.0–57.0) (*P* = .008) for breakthrough seizures.

Resource use and testing patterns differed by pathway use. Basic labs were ordered equally, but pathways prompted more extensive testing for common seizure-provoking factors, including urinalysis, alcohol level, and urine drug screen (*P* < .001). First seizure-pathway patients had significantly more head computed tomography (CT) than no-pathway patients (98.4 vs 52.8%, *P* < .001). Breakthrough seizure pathway patients had fewer MRI studies compared to standard-of-care patients (1.5 vs 9.1%, *P* = < .001). Use of EEGs did not differ (6.0 vs. 5.9%, *P* = .68).

## DISCUSSION

With this QI initiative we aimed to generate and implement standardized ED pathways for first seizure of life and breakthrough seizure. Pathway use led to faster outpatient access, reduced variability in ED LOS and diagnostic testing, and fewer hospital admissions. We hope these pathways provide a framework for other centers to optimize seizure care delivery in the ED.

### Access to Outpatient Care

Neurology access in the US is limited, with average wait times of up to 60 days, and up to six months for specialist care, which can result in delayed treatment, testing, and counseling.^16^,^17^–^19^,^20^,^21^ In our initiative, pathway patients, with or without an established primary care physician, saw neurologists significantly sooner. Institutions adopting seizure pathways should consider linking ED discharge to central scheduling to trigger expedited outpatient follow-up. Additionally, establishment of an associated outpatient clinic for first seizure evaluation can provide a direct outlet for expedited patient care and ensure the pathway is sustainable.^7^ While delays between ED discharge and outpatient visits persist, providing standardized seizure-safety literature can improve knowledge and confidence and bridge this gap.^22^ We did not directly assess these outcomes, which is a possible direction for future study.

### Decreased Length of Stay

Although median ED LOS was similar between groups, pathway use markedly reduced extreme ED LOS values (some over 24 hours), improving ED throughput and patient flow, which can improve patient satisfaction. Pathway usage resulted in significantly fewer hospital admissions. If repeated in larger cohorts, this finding could suggest meaningful reductions in healthcare costs and improve bed availability for other patients requiring urgent care.^23^–^25^

### Standardization of Care

Pathways promoted a more uniform evaluation of provoked vs unprovoked seizures, standardizing testing for common provoking factors (ie, urinalysis, urine drug screen, and alcohol level). They reduced MRI use in breakthrough seizures while ensuring that CT was completed for patients with first seizure of life, consistent with best practice guidelines. It is critical not to miss a structural lesion in this patient population as it is associated with a significantly higher risk of subsequent seizures, warranting more timely treatment with an anti-seizure medication. Use of EEG remained low and comparable between groups, aligning with evidence suggesting the low yield of a routine EEG in the ED, which thus could be deferred to an outpatient setting.^26^

## LIMITATIONS

This initiative had several limitations. The pathways were implemented in a single, large, academic health system, and further evaluation is needed to determine whether results are reproducible. Pathways were only used in a small proportion of seizure encounters. We found that a major barrier was EHR design, which prioritizes frequently used orders over order sets, requiring additional clicks, thereby reducing pathway use.^27^ Stronger behavioral nudges could improve adoption but were not feasible in this initiative. Additionally, while there was support from ED leadership and faculty, house staff, who often place the orders in the ED, were not directly targeted for education, highlighting a key area for improvement in future implementation efforts.

Pathway use was not randomized but at the discretion of the emergency physician. While this increases the fidelity of use in the natural environment, it can result in selection bias. Patient identification based on *ICD-10* codes are restrictive and do not distinguish between provoked, unprovoked, first, or breakthrough seizures. Additionally, we excluded patients with an *ICD-10* code of status epilepticus, which limited the patient population. Some of the granularity of a first seizure or breakthrough seizure, particularly in the non-pathway group, could have been lost due to incomplete *ICD-10* coding.

Future studies should evaluate pathways in larger, randomized cohorts and explore additional outcomes such as anti-seizure medication prescribing, adherence, seizure frequency, yield of ED neuroimaging, and cause of breakthrough seizures, as well as ease of use.[Table t2-wjem-27-61]

## CONCLUSION

Overall, seizure pathways improved efficiency, reduced unnecessary variation, and enhanced access to timely outpatient care, supporting their role as a scalable model for ED seizure management.

## Supplementary Information



## Figures and Tables

**Table 1:** Patient demographics of the pathway and standard-of-care groups in a study of the development and implementation of seizure care pathways in the emergency department.

	Standard of care	Pathway			

		Breakthrough seizure	First seizure of life	Pathway total	
Sex (n, %)					*P* = .14
Male	1,215 (45.6%)	38 (56.7%)	36 (57.2%)	74 (56.9%)	
Female	1,319 (49.5%)	29 (43.3%)	27(42.8%)	56 (43.1%)	
Age (median, IQR)	41 (30,57)	41 (25,534)	37 (29,49)	39 (29,51)	*P* =.09
Race (n, %)					*P* =.08
White	695 (26.1%)	8 (11.9%)	24 (38.0%)	31 (23.8)	
Black	1,614 (60.8%)	55 (82.1%)	33 (52.3%)	88 (67.7%)	
Asian	39 (1.4%)	0	4 (6.3%)	4 (3.1%)	
Other	226 (8.8%)	4 (6.0%)	2 (3.2%)	6 (4.6%)	

There was no significant difference between the demographics of the pathway group or the standard-of-care group.

**Table 2 t2-wjem-27-61:** Comparison of resource use between the standard of care, first seizure of life pathway, and breakthrough seizure pathway in a study of the development and implementation of seizure care pathways in the emergency department.

Ordered (n, %)	Standard of care	Breakthrough seizure	First seizure of life	Significance testing
MRI	232 (9.1%)	1 (1.5%)^a^	4 (6.4%)	*P* < .001
CT	1,340 (52.8%)	40 (59.7%)	62 (98.4%)^a^	*P* < .001
EEGa	151 (6.0%)	4 (6.0%)	3 (4.8%)	*P* = .68
Urinalysis	638 (25.1%)	40 (59.7%)^a^	36 (57.1%)^a^	*P* < .001
Urine drug screen	760 (29.9%)	45 (67.2%)^a^	51 (81.0%)^a^	*P* < .001
Ethanol level	525 (20.7%)	53 (79.1%)^a^	58 (92.1%)^a^	*P* < .001
CBC	2,145 (84.7%)	63 (84.7%)	63 (100%)	*P* <.001
BMP and/or CMP	2,158 (85.2%)	56 (107.9%)*	68 (100.1)%*	*P* <.001
Outcomes				
Length of stay	5.0 hours	4.6 hours	5.0 hours	*P* = .34
Admissions	120 (4.5%)	0 (0%)^a^	0 (0%)^a^	*P* < .001
Observation	96 (3.6%)	3 (2.3%)	5 (3.8%)	*P* = .55
Median readmissions (30 days)	1	1	1	*P* = .31
Median time to outpatient follow-up	41 days	24 days^a^	16 days^a^	*P* = .001

* Percentage is over 100% as both a BMP and CMP may have been ordered

a Denotes significant change from standard-of-care group.

*BMP*, basic metabolic panel; *CBC*, complete blood count; *CMP*, comprehensive metabolic panel, *CT*, computed tomography; *EEG*, electroencephalogram; *MRI*, magnetic resonance imaging.
