# Supplementary material for: Improving Standardization and Access to Care via Seizure Pathways in the Emergency Department
Source: West J Emerg Med. 2026 Jan 3;27(1):61–6. doi: 10.5811/westjem.48847 (PMC12815501; doi:10.5811/westjem.48847)
Supplement: Supplementary file 1 [file wjem-27-61-s001.docx]

**Supplemental Material 1**: **Seizure: Care Instructions**

Seizures are caused by abnormal patterns of electrical signals in the brain. They are different for each person.

Seizures can affect movement, speech, vision, or awareness. Some people have only slight shaking of a hand and do not lose consciousness. Other people may lose consciousness and have shaking of the whole body. Some people appear to stare into space. They are awake, but they can't respond normally. Later, they may not remember what happened.

A seizure may occur only once, or you may have them more than one time. Taking medicines as directed and following up with your doctor may help keep you from having more seizures. You may need additional tests to identify the type and cause of the seizures.

The doctor has checked you carefully, but problems can develop later. If you notice any problems or new symptoms, **get medical treatment right away.**

**Follow-up care is a key part of your treatment and safety.** Be sure to make and go to all appointments, and call your doctor if you are having problems. It's also a good idea to know your test results and keep a list of the medicines you take.

**How can you care for yourself at home?**

• Be safe with medicines. Take your medicines exactly as prescribed. Call your doctor if you think you are having a problem with your medicine.

• Inform family members and coworkers of your seizures and instruct them on appropriate first aid

• Avoid open fires or carrying hot pans; cook with other people present as much as possible

• Avoid climbing stairs or ladders, especially when alone

• Take showers instead of baths to avoid risk of drowning

• Use safety guards on equipment for cutting, chopping, and drilling

• Be sure that anyone treating you for any health problem knows that you have had a seizure and what medicines you are taking for it.

• Identify and avoid things that may make you more likely to have a seizure. These may include lack of sleep, alcohol or drug use, stress, or not eating.

• Avoid swimming alone, wear a life vest if possible

• Wear head protection when playing contact sports or when there is a risk of falling

• Carry two extra doses of medication in your purse or wallet in the event you forget medication while traveling

**Driving**

• You may not drive in Pennsylvania or New Jersey unless you have been seizure free for at least 6 months.

• Always wear a seatbelt

**Parenting Tips**

• Childproof your home as much as possible

• Dress, change, and sponge bathe the baby on the floor if doing so unattended

• Feed the baby while he/she is seated in an infant seat, if you are breastfeeding, sit on the floor with your back supported

• Keep a young baby in a playpen when you are alone

• Do not bathe your child unattended

• Do not be the only adult present when children are swimming

**When should you call for help?**

**Call 911** anytime you think you may need emergency care. For example, call if:

• If a seizure lasts longer than 5 minutes or two seizures occur in succession

• You have new symptoms, such as trouble walking, speaking, or thinking clearly.

• If the person having a seizure is choking or cannot breathe

**Call your doctor now** or seek immediate medical care if:

• You notice any changes in behaviors

Watch closely for changes in your health, and be sure to contact your doctor if you have any problems.

**Seizure First Aid Tips**

1) Always stay with the person until the seizure Is over

2) If able, time the seizure

4) Prevent injury by moving nearby objects out of the way

6) Do not forcibly hold the person down

7) Do not put anything in the person's mouth!

9) If the person is lying down, turn them on their side, with their mouth pointing to the ground. This prevents saliva from blocking their airway and helps the person breathe more easily.

10) If a person appears to be choking, turn them on their side and call for help. If they are not able to cough and clear their air passages on their own or are having breathing difficulties, call 911 immediately.

**Where can you learn more?**

**Go to https://www.epilepsy.com**

You and/or your family can get certified in seizure first aid by visiting https://www.epilepsy.com/living-epilepsy/seizure-first-aid-and-safety/first-aid-seizures-stay-safe-side

**How to schedule an appointment with the Penn Epilepsy Center?**

In certain cases, you may continue to experience symptoms related to your seizure or require ongoing specialized care. For additional evaluation or diagnostic testing, schedule an appointment with the Penn Epilepsy Center by calling 800-789-7366 or visiting pennmedicine.org/epilepsy.

Current as of: June 17, 2021 Content Version: 12.9

Copyrighted material adapted with permission from Healthwise, Incorporated

Care instructions adapted under license by Penn Medicine, Philadelphia, PA 800-789-PENN © 2011, The Trustees of the University of Pennsylvania. If you have questions about a medical condition or this instruction, always ask your healthcare professional. Healthwise, Incorporated disclaims any warranty or liability for your use of this information.

**Supplemental Material 2: CLONAZEPAM ORAL DISINTEGRATING TABLETS INFORMATION**

Clonazepam (Klonopin) is a medication used to treat seizures. The oral disintegrating tablet is used as a medication to help stop a seizure or prevent further seizures from occurring right after one has occurred.

***HOW SHOULD I TAKE MY MEDICATION?***

Clonazepam oral disintegrating tablets are to be administered if safe to do so during a seizure to help stop a seizure, or after a seizure has completed to prevent seizures from recurring. Make sure your hands are dry prior to touching the tablet. Peel the foil back in the pack (do not open the foil until you are ready to administer the medication). Do not push the tablet through the foil. Take one tablet and place it on the tongue of the patient who is having a seizure or who has completed their seizure, being careful to not place it too deep in their throat. The medication will dissolve with the patient’s saliva. Please call your doctor if you take your clonazepam oral disintegrating tablet. Do not take extra doses of this medication unless directed to do so by your physician, as an overdose of this medication can lead to excessive drowsiness, which can be extremely dangerous.

***CAN I TAKE CLONAZEPAM WITH OTHER MEDICATIONS?***

Yes, clonazepam may be taken with other prescribed medications. It may cause excessive drowsiness if combined with other medications or medications which also cause drowsiness (e.g., antidepressants, alcohol, antihistamines, pain medication).

***WHAT SIDE EFFECTS CAN BE CAUSED BY CLONAZEPAM?***

Just like other medications, clonazepam can cause side effects, but most patients do not have any significant side effects. If you experience any side effects that are bothersome, report them to your doctor. (There is a risk of serious, life-threatening side effects with all medications that are used to treat seizures. However, this risk is extremely small, and uncontrolled seizures can also be life-threatening.) The most common side effects that are experienced are:

• Sedation and incoordination. These side effects usually go away as your body adjusts to the medication or the dose is adjusted.

• Behavioral problems including depression or hyperactivity may occur.

• Some patients experience drooling from an increase in saliva. This usually improves with adjustment of the medication dose.

Women who plan to have children should consult with their doctor about the possible effects on the unborn child.

All antiepileptic medications are associated with an increased risk of thoughts of suicide. If you are concerned about thoughts of harming yourself or harming someone else, contact your physician or call 911 immediately.

If you have any questions or problems, please contact your doctor:
